# Tongxinluo Improves Cardiac Function and Ameliorates Ventricular Remodeling in Mice Model of Myocardial Infarction through Enhancing Angiogenesis

**DOI:** 10.1155/2013/813247

**Published:** 2013-08-27

**Authors:** Wen-Wu Bai, Yi-Fan Xing, Bo Wang, Xiao-Ting Lu, Ying-Bin Wang, Yuan-Yuan Sun, Xiao-Qiong Liu, Tao Guo, Yu-Xia Zhao

**Affiliations:** ^1^Key Laboratory of Cardiovascular Remodeling and Function Research, Shandong University, Jinan, Shandong 250012, China; ^2^Department of Traditional Chinese Medicine, Qilu Hospital, Shandong University, No. 107, Wen Hua Xi Road, Jinan, Shandong 250012, China

## Abstract

*Background*. Myocardial infarction (MI) is a major cause of morbidity and mortality in the world. Tongxinluo (TXL) is a traditional Chinese compound prescription which has cardioprotective functions. The present study was aimed to determine the effect of TXL on postischemic cardiac dysfunction and cardiac remodeling and to elucidate the underlying mechanisms. *Methods and Results*. MI was performed by ligation of left anterior descending coronary artery (LAD) in male adult mice. Mice were randomly divided into four groups: (1) sham group (Sham); (2) MI-control group (Control); (3) MI-low dose TXL group (TXL-L); and (4) MI-high dose TXL (TXL-H) group. Compared with the control group, TXL treatment restored cardiac function, increased revascularization, attenuated cardiomyocyte apoptosis, and reduced interstitial fibrosis. TXL treatment increased the phosphorylation of Akt, extracellular signal regulated kinase (ERK), and endothelial nitric oxide synthase (eNOS); the expression of phosphatidylinositol3-kinase (PI3K), hypoxia-inducible factors 1**α** (HIF-1**α**), and vascular endothelial growth factor (VEGF); and the DNA binding activity of HIF-1**α** after MI. *Conclusion*. TXL may improve cardiac function and ameliorate cardiac remodeling by increasing neovascularization through enhancing the phosphorylation of Akt and ERK, the expression and activity of HIF-1**α**, and the protein level of VEGF and p-eNOS.

## 1. Introduction

Myocardial infarction (MI) is a major cause of morbidity and mortality. It remains responsible for about one-third of heart failure cases in our world, although there are lots of therapeutic approaches [1]. In fact, sudden occlusion of a major coronary artery can result in acute myocardial ischemia and rapid apoptosis of cardiomyocytes, which leads to progressive fibrous replacement of myocardium and left ventricular (LV) dilatation [2]. The progressive deterioration LV remodeling contributes to post-MI heart failure, and the prognosis of patients with heart failure is still poor [3]. Therefore, targeting promotion of new vessels to increase blood flow to ischemic tissues is a promising option to treat ischemic heart disease [4].

Tongxinluo (TXL) is a traditional Chinese compound prescription and has been approved by the State Food and Drug Administration of China in 1996 for the treatment of angina pectoris and ischemic stroke, as we previously described [5]. Increasing evidence has indicated that the traditional Chinese medicine tongxinluo has cardioprotective functions. Treatment with tongxinluo is effective in lowering serum lipid levels, inhibiting plaque inflammation, and enhancing stability of vulnerable plaques [5]. It can also reduce myocardial no-reflow and ischemia-reperfusion injury [6] and modulate vascular endothelial function [7].

However, it remains unclear whether TXL has an effect on cardiac function and heart remodeling after myocardial infarction. Therefore, we designed this experiment to investigate the potential role and mechanism of TXL in mice model of MI by ligation of anterior descending branch (LAD).

## 2. Material and Methods

### 2.1. Experimental Animal

The animal experimental protocol complied with the Animal Management Rules of the Chinese Ministry of Health (document number 55, 2001) and was approved by Animal Care Committee of Shandong University. The study including all animals was carried out at the Animal Care Center of Key Laboratory of Cardiovascular Remodeling and Function Research, Shandong University. Ten-week-old male C57BL/6 background wild-type mice (purchased from Vital River Laboratories, Beijing, China) were used for the study. The mice were fed regular mice chow and housed in a normal night-day rhythm under standard conditions of temperature and humidity.

### 2.2. Preparation of Tongxinluo Ultrafine Powder Solution

Tongxinluo ultrafine powder (Lot number 120109, Shijiazhuang Yiling Pharmaceutical, Co., Shijiazhuang, China) was dissolved in physiological saline at the concentration of 150 mg/mL and 38 mg/mL for this study. Fresh suspensions of TXL superfine powder were prepared in darkness each day. The herbal drugs were authenticated and standardized against marker compounds according to the Chinese Pharmacopoeia (2005).

### 2.3. Animal Grouping

Ten-week-old male wild-type mice were randomly divided into four groups: (1) sham group (Sham); (2) MI-control group (Control), where the mice were treated with vehicle (physiological saline) alone (10 mL/kg/day); (3) MI-low dose TXL group (TXL-L), where mice were treated with 0.38 g/kg/day TXL; (4) MI-high dose TXL (TXL-H) group, where mice were treated with 1.5 g/kg/day TXL. The mice were either subjected to left anterior descending coronary artery ligation (LAD) or the same time-matched surgical procedure without ligation. 

### 2.4. Surgical Procedures

MI was induced in experimental animals as described [8]. All animal surgeries were carried out under isoflurane (2%) to minimize the pain. Mice were orally intubated and artificially ventilated by using a rodent respirator (Harvard Apparatus, USA). The tidal volume was set at 250 *μ*L, and the respiratory rate was set at 120 breaths per minute. Hearts were then exposed through the left lateral thoracotomy. MI was created by permanent LAD ligation with a 7–0 suture line. The occlusion of coronary was confirmed by pallor and regional wall motion abnormality of the left ventricle. The sham group underwent the same time-matched surgical procedure without ligation.

### 2.5. Echocardiography

All measurements represented the mean of at least three consecutive cardiac cycles. Transthoracic echocardiography was performed in mice 30 days after MI surgical intervention to evaluate left ventricle function. We removed the hair on the chest wall and spread ultrasound gel over the precordial region of the isoflurane anesthetized mice. Cardiac dimensional and functional parameters were analyzed by using a high resolution echocardiography system (Vevo 770, Visual Sonics) with a 35 MHz linear array transducer. M-mode echocardiography of the left ventricle at the papillary muscle level was performed. Diastolic left ventricular internal diameter (LVIDd) and systolic left ventricular internal diameter (LVIDs) were measured on the M-mode tracings. The left ventricular fractional shortening (%FS) and ejection fraction (%EF) were automatically calculated by the echocardiographic system.

### 2.6. Histological Analysis

Histological analysis was assessed in perfusion/fixed hearts collected from mice at 7 days or 30 days after surgery (*n* = 5-6 mice/group). Briefly, the mice were euthanized by pentobarbital (50 mg/kg). The chest was opened and the heart was arrested in diastole by intraventricular injection of KCL (10%). The right atrium was then cut and the myocardial vasculature was perfused, followed by 10 min perfusion with 10% formalin. The hearts were harvested and fixed in 4% formalin for 24 hours. The formalin-fixed tissues were embedded in paraffin wax and cut into 5 *μ*m sections for hematoxylin-eosin and Masson's trichrome staining. The LV endocardium was traced, and the endocardial circumference was calculated. Infarct size was expressed as the sum of the epicardial and endocardial scar length divided by the sum of the LV epicardial and endocardial circumferences [9]. Masson's trichrome staining was used to detect interstitial fibrosis in the border zones. Peri-infarct (border-zone) region was defined as the 2 mm area encircling the area of pathologic infarction [9]. Collagen volume fraction (CVF) of the peri-infarct zone was calculated as the ratio of Masson's trichrome-stained collagen area to the border zone myocardium area, as described [3]. The morphologic parameters and CVF were measured using Image-Pro Plus 6.0, by two blinded observers.

### 2.7. TUNEL Assay

The apoptosis of cardiomyocytes was assessed by using In Situ Cell Death Detection Kit (Millipore, MA, USA) according to the manufacturer's specifications. The percentage of TUNEL-positive cardiomyocytes was counted. Five fields of apoptotic positive cells were counted under 400x magnification in a blinded fashion. Cells were defined as apoptotic if the whole nuclear area of the cell was labeled positively [10].

### 2.8. Capillary Density

For the measurement of capillary density (counts/mm^2^), we performed immunohistochemical analysis of CD31/Platelet Endothelial Cell Adhesion Molecule-1 (1 : 50; Santa Cruz Biotechnology, CA, USA). Transverse sections of the short axis of the left ventricle per sample were used in this analysis. Five fields on the slide were randomly chosen for counting the stained capillaries in the border zone between infarcted area and noninfarcted area (corresponding fields were selected in the sham group animals) at 400x magnification. All stained capillaries were counted, and the density was expressed as number/mm^2^ [8].

### 2.9. Western Blot Analysis

Protein in the border zone of heart tissues was extracted at 7 days after surgery. Protein of equal mounts was separated on 10%–15% SDS-PAGE and electrotransferred onto nitrocellulose membrane (Amersham Biosciences, NJ, USA). After blocked with 5% nonfat milk for 2 h at room temperature, blots were washed in TBS-T 3 times for totally 30 min and incubated with primary antibodies at 4°C room overnight. The primary antibodies were as follows: rabbit monoclonal polyclonal anti-GADPH (1 : 2000, Cell Signaling Technology, MA, USA), anti-phospho-ERK/ERK (1 : 1000, Cell Signaling Technology, anti- PI3K(1 : 1000, Cell Signaling Technology), anti-phospho-AKT (Ser473)/AKT (1 : 1000, Cell Signaling Technology), anti-polyclonal HIF-1*α* (1 : 500, Abcam), anti-polyclonal VEGF (1 : 500, Proteintech Group, Inc., Chicago, USA), anti-phospho-eNOS (Ser1177) (1 : 1000, Cell Signaling Technologies), and anti-eNOS (1 : 1000, Sigma, MO, USA). After being washed in TBS-T, membranes were incubated with horseradish peroxidase-conjugated secondary antibody for 2 h at room temperature. Signals were detected by enhanced chemiluminescence (Millipore) and analyzed by the use of Image-Pro Plus 6.0.

### 2.10. Electrophoretic Mobility Shift Assay (EMSA) Analysis

EMSA was performed by using LightShift Chemiluminescent EMSA Kit (Thermo Scientific, MO, USA) according to the manufacturer's instructions [11]. Briefly, nuclear protein in the border zone of heart tissues was collected and extracted by Nuclear Extraction Kit (Active Motif, CA, USA). Single-stranded oligonucleotides containing binding sites of HIF-1*α* (5′-GCCCTACGTGCTGTCTCA-3′) were synthesized and end-labeled with biotin (BioSune, Shanghai, China). Single-stranded oligonucleotides were annealed to double-strand probes for EMSA. Nuclear protein (10 *μ*g) was incubated with 20 fmol biotin-labeled oligonucleotides for 20 min at room temperature. The specificity of the HIF-1*α*-DNA binding was determined in competition reactions in which a 100-fold molar excess of unlabeled oligonucleotide was added. After electrophoresis in 6.5% PAGE, protein-oligonucleotide complex was electroblotted to a positively charged nylon membrane. Then transferred DNA was cross-linked to membrane with a UV lamp for 10 min. After incubation in blocking buffer for 30 min at room temperature, the membrane was washed and incubated with substrate equilibration buffer for 20 min at room temperature. The membrane was then incubated with chemiluminescent substrate for 5 min before being analyzed in Image Quant LAS 4000 mini (GE Healthcare).

### 2.11. Statistical Analysis

The data are expressed as mean ± SEM. SPSS for Windows v.18.0 (SPSS Inc., Chicago, IL, USA) was used for statistical analysis. Intergroup comparisons involved one-way ANOVA followed by LSD's test (with equal variances assumed) or Dunnett'sT3 test (with equal variances not assumed). Probabilities of 0.05 or less were considered to be statistically significant.

## 3. Results

### 3.1. TXL Treatment Improved Post-MI Cardiac Dysfunction

**Figure 1:**
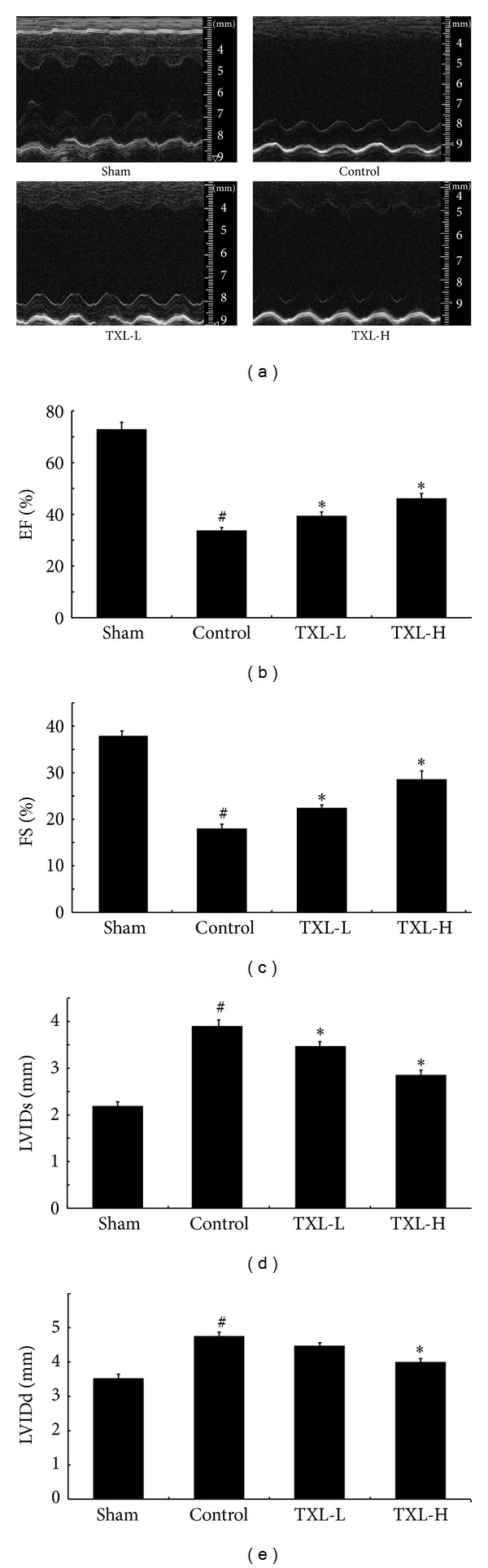
TXL treatment could improve the cardiac function as determined by echocardiography on the 30th day after surgical intervention. (a) Representative echocardiograph pictures of M mode images. The quantitative data of (b) ejection fraction (EF); (c) fractional shortening (FS); (d) left ventricular internal diameter in systole (LVIDs); and (e) left ventricular internal diameter in diastole (LVIDd). These results demonstrate that there was significant functional disorder in the control group compared with the sham group. TXL treatment significantly improved functional parameters compared with the animals after MI (*n* = 5-6 per group). ^#^ *P* < 0.05 versus Sham; *P < 0.05 versus Control.

Left ventricular functional parameters were studied by echocardiography at 30 days after coronary ligation. As shown in Figure 1, left ventricular function was preserved in TXL treatment groups as assessed by ejection fraction and fractional shortening in comparison with the control group. Meanwhile, diastolic left ventricular internal diameter (LVIDd) and systolic LVID (LVIDs) were both lower in the TXL-treatment animals than in those in the control group. Therefore, compared with the control group, TXL treatment could significantly improve the LV function.

### 3.2. TXL Therapy Attenuated Infarction Size after MI

**Figure 2:**
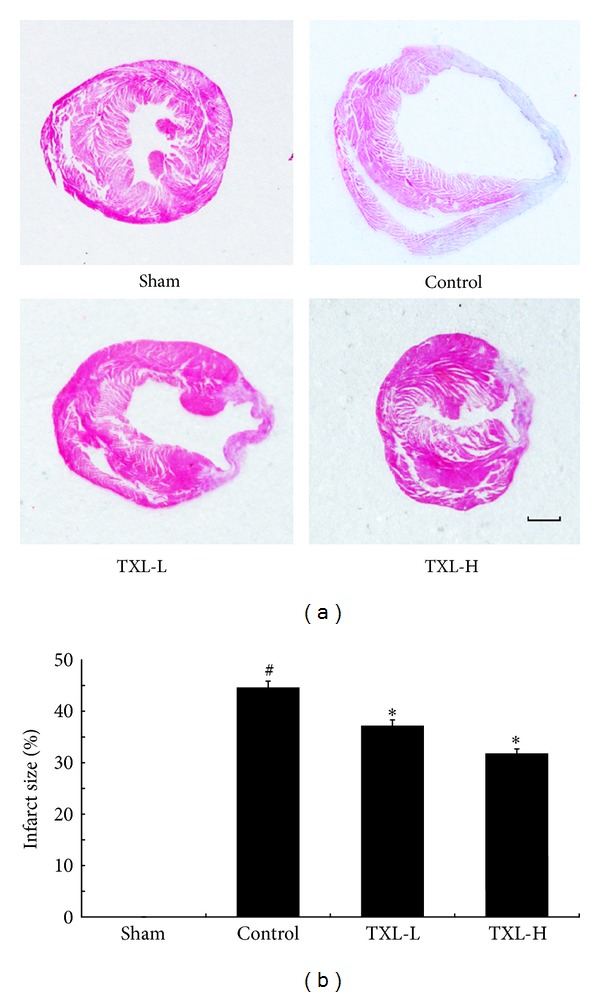
TXL treatment reduced infarction size after MI. (a) Paraffin-embedded sections of myocardium were stained with hematoxylin-eosin (bar = 1 mm). (b) Quantification of infarct size from four groups (*n* = 5-6). ^#^ *P* < 0.05 versus Sham; *P < 0.05 versus Control.

Hematoxylin-eosin staining was used to measure the infarct size in different groups. As shown in Figure 2, compared with the sham group, LAD ligation induced myocardial infarction, while TXL treatment could significantly reduce the size of infarction at a dose-dependently manner, which suggested that TXL had a protective effect against MI.

### 3.3. TXL Therapy Attenuated Myocardium Interstitial Fibrosis

**Figure 3:**
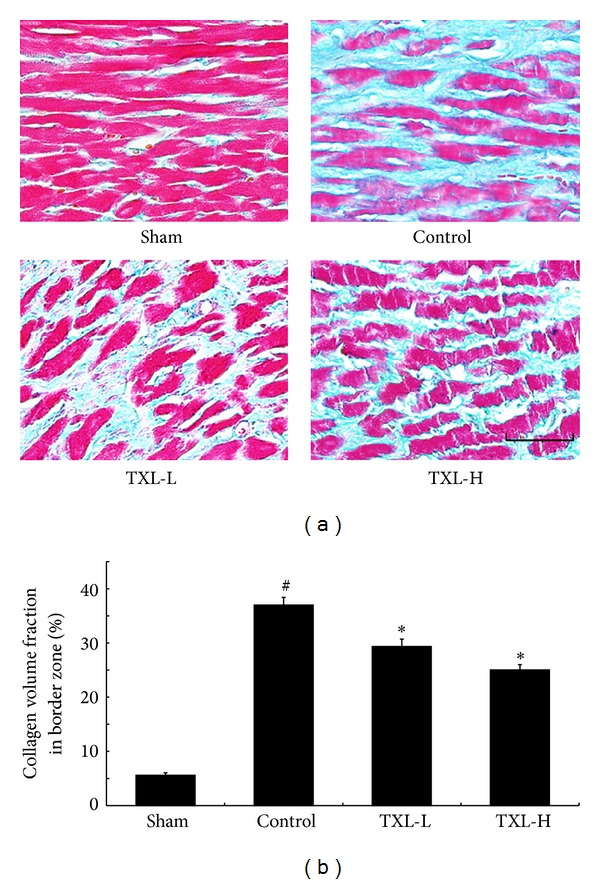
TXL treatment reduced interstitial fibrosis in the peri-infarct border zone 30 days after MI. (a) Masson's trichrome staining was performed to detect interstitial fibrosis in the border zone. (b) Quantification of interstitial fibrosis expressed as collagen volume fraction of border zone (*n* = 5-6). Bar = 50 *μ*m. ^#^ *P* < 0.05 versus Sham; *P < 0.05 versus Control.

30 days after MI, Masson's trichrome staining was performed to show the fibrosis in the border zone. As shown in Figure 3, there was seldom collagen in the sham group; LAD ligation significantly increased the collagen volume fraction (CVF) of border zone, while TXL treatment could significantly reduce it. The result suggested that TXL therapy could attenuate interstitial fibrosis induced by MI.

### 3.4. TXL Therapy Reduced MI-Induced Cardiomyocyte Apoptosis

**Figure 4:**
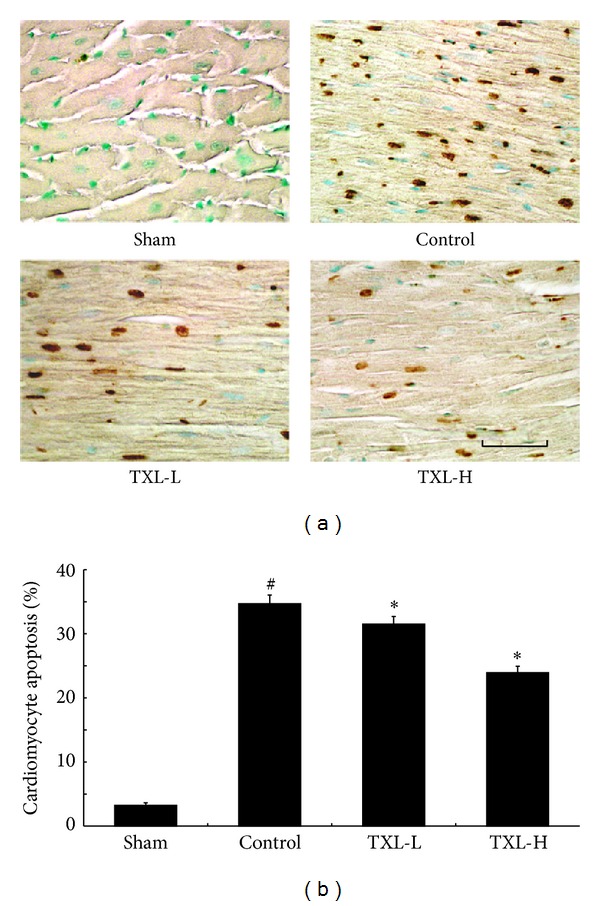
TXL treatment attenuated cardiomyocyte apoptosis after MI. (a) The apoptosis of cardiomyocytes was determined by TUNEL staining. (b) Quantitative analysis of cardiomyocyte apoptosis after MI from 5-6 animals. Bar = 50 *μ*m. ^#^ *P* < 0.05 versus Sham; *P < 0.05 versus Control.

The extent of cardiomyocytes apoptosis was detected by using TUNEL staining. In the border zone, there was seldom cardiomyocytes apoptosis in the sham group, while the percentage of TUNEL-positive cardiomyocytes was markedly reduced after TXL treatment as compared with the control group (Figure 4). These results demonstrated that TXL acted as an antiapoptotic agent during myocardial infarction.

### 3.5. TXL Therapy Increased Capillary Density

**Figure 5:**
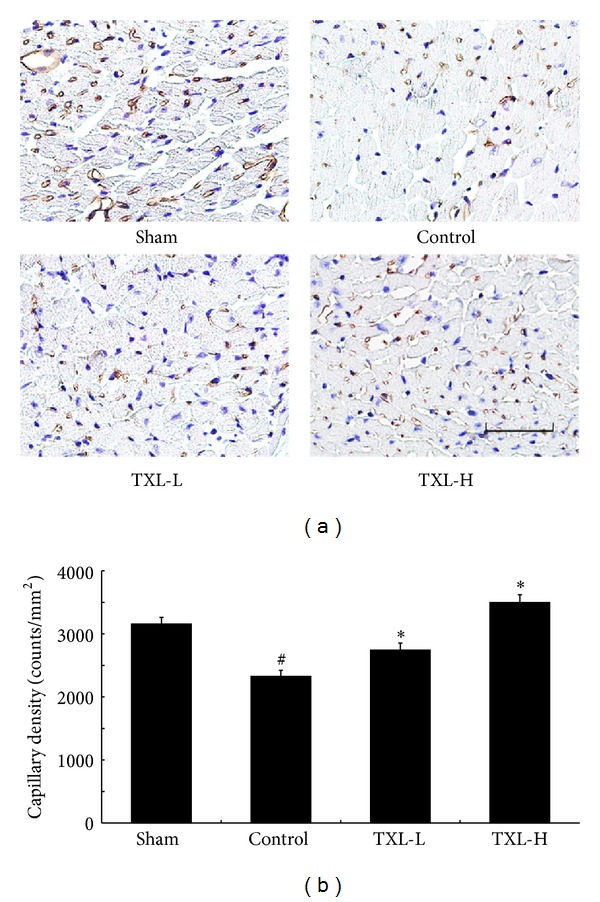
TXL therapy increased the neovascularization after 7 days of MI. (a) Representative digital micrographs showing capillary density/CD31 in different experimental groups. (b) Quantitative analysis of capillary density in counts/mm^2^. The values are mean ± SEM of 5-6 animals per group. Bar = 50 *μ*m. ^#^ *P* < 0.05 versus Sham; *P < 0.05 versus Control.

The role of TXL treatment on the degree of angiogenesis after myocardial infarction was measured by capillary density 7 days after surgical intervention. Compared with the control group, increased capillary was observed in the TXL treatment groups (Figure 5). These results demonstrated that TXL treatment promoted neovascularization by increasing capillary after myocardial infarction.

### 3.6. TXL Treatment Increased the Expression of PI3K, HIF-1*α*, and VEGF and Elevated Phosphorylation of AKT, ERK, and eNOS

**Figure 6:**
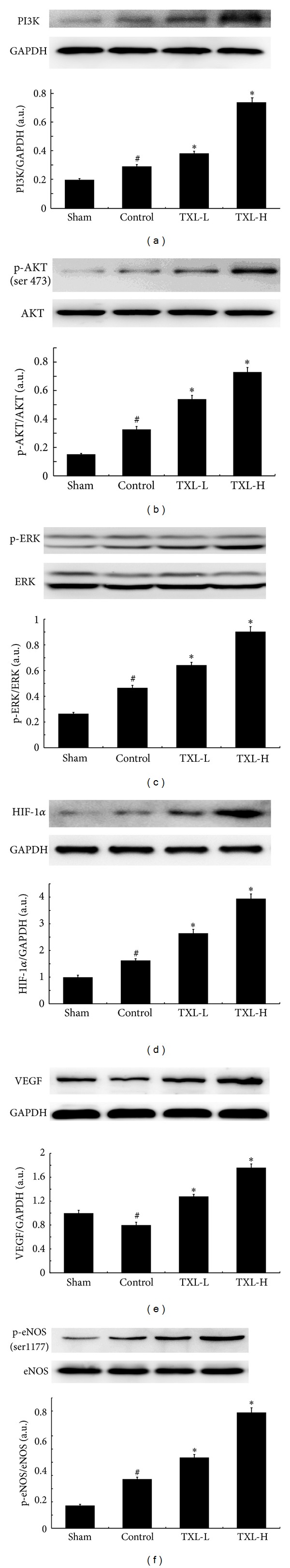
TXL treatment increased the expressions of PI3K, p-Akt, p-EKR, HIF-1*α*, VEGF, and p-eNOS.

Then we investigated the probable mechanism.  Figure 6 showed the higher intensity of PI3-kinase, phosphorylated Akt (p-Akt), and phosphorylated ERK (p-ERK) in the TXL groups compared with the control group. HIF-1*α*, VEGF, and phosphorylated eNOS (p-eNOS) protein levels were evaluated by western blotting. The levels of these proteins were increased in TXL therapy groups compared with the control group.

Representative western blots show the expression of (a) PI3K, (b) p-Akt, (c) p-ERK, (d) HIF-1*α*, (e) VEGF, and (f) p-eNOS. Bar graphs in panels (a), (b), (c), (d), (e), and (f) represent the quantitative analysis and difference in the expression of these proteins among different groups, after they were normalized with corresponding nonphosphorylated protein controls or their corresponding loading control-GAPDH, respectively, in arbitrary units (*n* = 3–5 from each group).

There was a significant increase in the expression of these proteins in TXL groups compared with the control group. ^#^ *P* < 0.05 versus Sham; *P < 0.05 versus Control.

### 3.7. TXL Treatment Increased DNA-Binding Activity of HIF-1*α*

**Figure 7:**
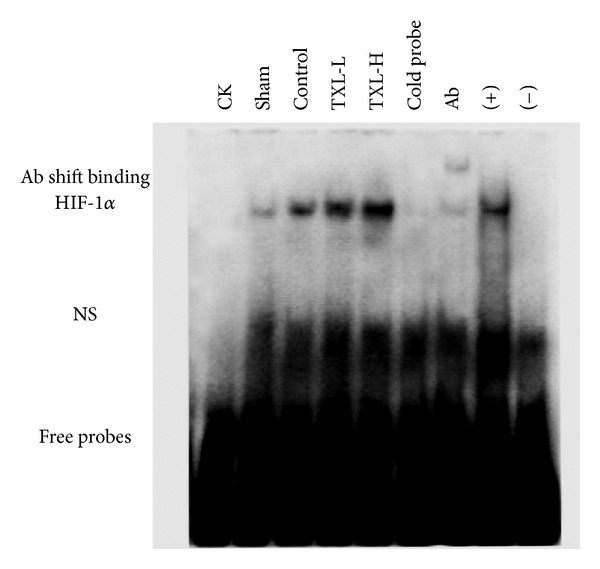
TXL treatment increased the HIF-1*α* DNA-binding activity after MI. HIF-1*α* DNA-binding activity was assessed by EMSA (*n* = 3 from each group). NS: nonspecific binding.

Then the effect of TXL on transcriptional activity of HIF-1*α* after myocardial infarction was evaluated by EMSA analysis. As shown in Figure 7, the DNA binding activity of HIF-1*α* was significantly increased in TXL treatment groups compared with control group, which suggested that TXL played a protective effect against MI by increasing the DNA-binding activity of HIF-1*α*.

## 4. Discussion 

In this study, we demonstrated that treatment with traditional Chinese medicine TXL dose dependently improved cardiac function and attenuated ventricular fibrosis and myocardial apoptosis by increasing neovascularization after MI. In further study, we found that TXL played a protective effect by enhancing the DNA-binding activity of HIF-1*α* and modulating the expression of VEGF and eNOS.

Traditional Chinese medicine (TCM) has a history of thousands of years and has made lots of contributions to the health of people. It has been reports that TCM plays an important role in treating cardiac dysfunction and LV remodeling after MI [10, 12–14]. Tongxinluo is a traditional Chinese medicine which is extracted, concentrated, and freeze-dried from a group of herbal medicine, such as ginseng, Radix Paeoniae Rubra, borneol, and spiny jujuba seed, which contains multiple active components that may be responsible for its antianginal effects as described [5, 15]. It has been widely used for coronary heart disease and unstable angina pectoris in China for nearly twenty years. Although it has been demonstrated that administration of TXL shows cardioprotective functions [5–7, 15], the effects of TXL treatment in MI mice have been unclear. In our experiment, we found that TXL had protective effects on cardiac function and ventricular remodeling after MI.

Myocardial infarction, which is mainly caused by atherosclerosis induced vascular stenosis, is a severe threat to human health. After myocardial infarction, abnormal myocardial blood flow can impaire myocardial O_2_ delivery, resulting in the maladaptive remodeling in left ventricular, including cardiac dysfunction, apoptosis, and fibrosis [2, 3]. There will be a lot of compensatory responses of the body after MI including spontaneous neovascularization, but it is not enough to satisfy the heart needs [8, 16]. Therapeutic angiogenesis is an attractive approach to cure or at least improve alleviate ischemic cardiovascular disease [4, 17, 18].

In our study, compared with the control group, administration of TXL promoted neovascularization by increasing capillary density. The increased vascular density might have resulted in increased regional perfusion, potentially accounting for the observed improvement in the left ventricular function and LV remodeling. Then we investigated the probable mechanism by which TXL could promote angiogenesis. Angiogenesis is the most important repair process of tissues subjected to ischemic insult, and stimulation of neovascularization is expected to reduce ventricular dysfunction and remodeling after MI [19].

The PI3-kinase/Akt signalling pathway has an important role in inducing vascularization of heart and inhibiting cardiomyocyte apoptosis after MI [20, 21]. Activation of the PI3K/AKT pathway leads to increased translation of HIF-1*α* mRNA and elevated HIF-1*α* protein levels [22]. The ERK MAP kinase pathway is also a potential signaling cascade in the modulation of angiogenesis and ERK signalling could reduce apoptosis in myocardium [23, 24]. It has been reported that hypoxia could result in ERK activation, which could further elevate the expression of HIF-1*α* and increase its activity by direct phosphorylation or indirect phosphorylation [25].

HIF-1*α*, a transcription factor, which is a major regulator of the hypoxic response of ischemia, plays a pivotal role in angiogenesis which can increase oxygen delivery [26–28]. Accumulating evidence shows that HIF-1*α* acts as potential therapeutic proangiogenic molecules in experimental models [9, 24]. Therapy based on expression of HIF-1*α* can be regarded as a strategy to induce neoangiogenesis in the ischemic heart. HIF-1*α* activity has been demonstrated to be under the regulation of protein kinase phosphorylation, including PI3K/AKT and ERK [29]. Interestingly, the protein kinases, Akt and Erk, have been both implicated as mediators of cardioprotection [30]. Numerous reports documented that HIF-1*α* directly influences the expression of VEGF and eNOS [31]. VEGF, an endothelial-specific mitogen and survival factor, is one of the most potent angiogenic factors, and it is a key factor for both angiogenesis and vasculogenesis [32]. eNOS can be activated in phosphorylation of serine 1177 residue and this triggers migration and angiogenesis [33, 34].

In the experiment, our results showed that compared with the control group, TXL treatment could significantly increase the expression of PI3K and phosphorylation of Akt as well as ERK, with an increased expression and DNA-binding activity of HIF-1*α*. Moreover, TXL treatment could also increase the expression of VEGF and phosphorylation eNOS, which subsequently led to enhanced angiogenesis and neovascularization.

## 5. Conclusion

In conclusion, the present study indicated that TXL therapy may dose-dependently improve the cardiac function and ameliorate ventricular remodeling after MI by inducing angiogenesis and neovascularization via enhancing the phosphorylation of Akt and ERK, expression and activity of HIF-1*α*, and the protein level of VEGF and p-eNOS. TXL may be an option for the treatment of cardiac dysfunction and ventricular remodeling after MI.
